# The microbiological profile of necrotising fasciitis at a secondary level hospital in Gauteng

**DOI:** 10.4102/sajid.v39i1.542

**Published:** 2024-04-15

**Authors:** Mbavhalelo C. Molewa, Agata Ogonowski-Bizos, Mariska Els, Cheryl M. Birtles, Molebogeng C. Kolojane

**Affiliations:** 1 Department of Surgery, Edenvale Regional Hospital, Johannesburg, South Africa; 2 Department of Surgery, Faculty of Health Sciences, University of the Witwatersrand, Johannesburg, South Africa; 3 Infection Control Services Laboratory, National Health Laboratory Services, Charlotte Maxeke Johannesburg Academic Hospital, Johannesburg, South Africa; 4 Department of Clinical Microbiology and Infectious Disease, Faculty of Health Sciences, University of the Witwatersrand, Johannesburg, South Africa

**Keywords:** necrotising fasciitis, polymicrobial, monomicrobial, antimicrobial sensitivity, antimicrobial resistance

## Abstract

**Background:**

Necrotising fasciitis (NF) is a fulminant soft tissue infection that requires timely diagnosis, urgent surgical debridement, and appropriate antimicrobial therapy. The choice of empiric antimicrobial therapy depends on the microorganisms cultured and the antimicrobial resistance profile of the institution. Necrotising fasciitis has not been studied in our setting.

**Objectives:**

The aim of the study was to audit the microbiological profile of NF and antimicrobial susceptibility profile.

**Method:**

This was a retrospective study in a secondary level hospital from the period of 2014–2020. The patients’ demographic data, clinical features, location of infection, comorbidities, laboratory and microbiological profiles were analysed.

**Results:**

There were 53 patients during 2014–2020 with median age of 45.5 (38.5–56.0) years. The majority of the patients were males (35 [66.04%]), had no comorbidities (25 [47.17%]), and the lower limb was the most common anatomic site (17 [32.08%]). Type II (monomicrobial) NF was the predominant type (31 [58.49%]). *Staphylococcus aureus* was the most prevalent Gram-positive bacteria (18 [38%]) and *Escherichia coli,* the main species isolated in the Gram-negative bacteria (14 [36%]) with susceptibility to cloxacillin (94%) and amoxicillin and/or clavulanic acid (92%), respectively.

**Conclusion:**

*Staphylococcus aureus* and *Escherichia coli* were the most common bacteria with low rate of antimicrobial resistance. Amoxicillin and/or clavulanic acid and an adjunctive clindamycin are appropriate antimicrobial therapy for empiric treatment for NF in our setting.

**Contribution:**

Amoxicillin and/or clavulanic acid and an adjunctive clindamycin can be used as an empiric treatment for NF.

## Introduction

Necrotising fasciitis (NF) is a fulminant soft tissue infection that spreads rapidly along the fascial planes, and when unabated can lead to septic shock and death.^1^ It is a rare soft tissue infection with reported incidence of 0.3–3/100 000 persons per year with a mortality rate of up to 32% in Asia and Europe.^2,3^ Necrotising fasciitis can affect any part of the body; lower limbs (32%), upper limbs (24%), perineum (16%), trunk (16%), and head and neck (10%).^1^ The commonly reported risk factors associated with NF are diabetes, alcoholism, chronic kidney disease, and liver cirrhosis.^2^

The clinical presentation of NF can overlap with clinical features of other soft tissue infection such as cellulitis. Wong et al.^4^ reported a misdiagnosis of 73% of NF cases as cellulitis and subsequently developed Laboratory Risk Indicator for Necrotising Fasciitis (LRINEC) score to aid in distinguishing NF from other soft tissue infections. The score is calculated from six parameters: C-reactive protein, total white cell count, haemoglobin, sodium, creatinine, and glucose.^4^

There are four types of NF classified according to the microorganisms often isolated in culture of NF infections. Type I is a polymicrobial infection including, Group A *Streptococcus* (GAS), *Bacteroides fragilis, Staphylococcus aureus, Clostridium perfringens* and *Enterobacterales*. Type II infections are monomicrobial mainly the Group A *Streptococcus* or methicillin-resistant *Staphylococcus aureus* (MRSA). Type III is the marine *Vibrio vulnificus* species that inoculate injuries exposed to sea water.^1^ Type IV is an uncommon type of NF caused by fungal infection.^5,6^

Once a diagnosis or suspicion of NF is made, broad-spectrum antimicrobial treatment is commenced and surgical debridement is performed. Early surgical debridement and an appropriate antimicrobial therapy is crucial to improve survival outcomes.^7^ Antimicrobial resistance has emerged to be a threat to public health. In 2019, Antimicrobial Resistance Collaborators estimated 4.95 million (3.62–6.57) deaths associated with bacterial antimicrobial resistance. Sub-Saharan regions have been found to have the highest mortality associated with antimicrobial resistance at 27.3 per 100 000 (20.9–35.3). The common six pathogens associated with antimicrobial resistance deaths were: *Pseudomonas aeruginoasa, Escherichia coli,* MRSA, *Klebsiella pneumoniae, Streptococcus pneumoniae* and *Acinetobacter baumannii. Staphylococcus aureus* alone was responsible for 100 000 deaths associated with antimicrobial resistance in 2019.^8^ There has been emerging reports of multidrug resistance *Acinetobacter baumannii* skin and soft tissue infections across different parts of the world. Of concern, is that some of the multidrug resistance *Acinetobacter baumannii* skin and soft tissue infections were reported in previously healthy patients.^9,10,11^

Necrotising fasciitis is a fulminant soft tissue infection that has not been well studied in South Africa. There were only a handful of case reports published on NF in a South African setting. These cases of NF were associated with treatment; immune suppressive therapy, radiation therapy, and diclofenac intramuscular injection.^12,13,14^ It is imperative to grow a body of knowledge on this subject. The cornerstone of treatment in NF is surgical debridement and antibiotics. Amoxicillin and/or Clavulanic acid and clindamycin are the antibiotic of choice in our institution; however, this choice was not informed by prior clinical research. Antimicrobial resistance profile in NF infections had not been studied in our setting. The primary objective was to describe the microbiology profile of NF and to describe the antimicrobial susceptibility thereof. The secondary objectives were to describe the clinical and laboratory findings of NF, to determine the comorbidities and risk factors associated with NF, and to describe the LRINEC score in the patients with NF.

The results of this study will help inform antimicrobial protocols in the Antimicrobial Stewardship Committees in the institution and in other institutions like ours. There is no doubt that studies on NF are very much needed in South Africa to fill up the existing gap in the literature.

## Methods and design

### Study design and setting

This was a retrospective review of patients records at Edenvale Regional Hospital of Gauteng province in South Africa. Edenvale Regional Hospital is a secondary hospital that receives patients from local primary healthcare facilities and private general practices. It refers critically ill patients to a nearby tertiary hospital for intensive care and complex surgical management.

### Study population

All patients of age 18 years and above, who were admitted between the period of 2014 and 2020 with a diagnosis of NF and had a surgical debridement for NF were included in the study. Patients who neither had surgical debridement nor specimen sent for microbiology analysis, were excluded from the study.

### Data collection

Patients’ records were retrieved from hospital archives. Clinical notes, operative notes, and treatments charts were critically reviewed. Microbiology and blood results were requested from National Health Laboratory Service. Excel worksheet was used to capture relevant information from the patients’ clinical records. The following variables were extracted: demographics, comorbidities, risk factors, site of infection, intra-operative findings, blood and microbiology results. Laboratory Risk Indicator for NF score was calculated using C-reactive protein, white blood cell, haemoglobin, sodium, creatinine, and glucose, and was classified into three categories: low risk (score ≤ 5), moderate risk (score 6–7) and high risk (score ≥ 8).^4^

### Data analysis

Data were analysed with StataCorp 2021 (Stata Statistical Software: Release 17. College Station, TX: StataCorp LLC). Continuous variables were expressed as means and medians and categorical variables were expressed as frequencies and percentages. The comparative analysis was performed to establish correlation between the NF microbiology type and demographics and laboratory results using parametric and non-parametric tests; independent *t*-test, Mann–Whitney test, one way analysis of variance (ANOVA), Kruskal–Wallis and Fisher’s exact test. A *p*-value of less than 0.05 was considered statistically significant.

### Ethical considerations

Ethical approval for the study was given by the University of the Witwatersrand Human Research Ethics Committee (M210513), Johannesburg Health District Research Committee (GP 202106-038) and National Health Laboratory Services (PR2117899). Informed consent was not required for retrospective review of patients’ records.

## Results

A total of 65 patients were admitted with NF to Edenvale Hospital between January 2014 and December 2020. A total of 12 patients were excluded from the study; 4 patients had no culture sent for microscopy, 3 patients’ cultures had no growth, and 5 patients had incomplete operation notes. A total of 53 patients were included in the study. The median age of the patients was 45.5 (38.5–56) years and majority of the patients were males (35 [66%]). Many of the patients had no comorbidities reported (25 [47.17%]). Necrotising fasciitis affected mainly the lower limbs (17 [32.08%]) with a low risk LRINEC score of 5 or less (13 [59.09%]) (Table 1). The LRINEC score analysis was underpowered as only 22 patients had all the parameters for LRINEC score calculation.

**TABLE 1:** Demographics, clinical and laboratory characteristics of the patients.

Characteristics	Number of patients	Median	%
**Demographics**			
Age in years	45.5	38.5–56	-
Male gender	35	-	66.04
Female gender	18	-	33.96
**Comorbidities**			
HIV	16	-	30.19
Diabetes	12	-	22.64
None	25	-	47.17
**Anatomic site**			
Trunk	14	-	26.42
Perineum and scrotum	12	-	22.64
Buttocks	5	-	9.43
Upper limb	4	-	7.55
Lower limbs	17	-	32.08
Head and neck	1	-	1.89
**Intra-operative findings**			
Necrotic tissue	53	-	100.00
Pus	24	-	45.28
Crepitus	1	-	0.02
**LRINEC categories**			
5 or less	13	-	59.09
6–7	3	-	13.64
8 or more	6	-	27.27
**Type of NF**			
Type I	21	-	39.62
Type II	31	-	58.49
Type III	0	-	0.00
Type IV	1	-	1.89
**Laboratory results**			
C-reactive protein mg/L	181	95–290	-
White cell count, per mm^3^	13.42	8.6–19.4	-
Haemoglobin, g/L	9.55	8.2–11.8	-
Sodium, mmol/L	136	134–139	-
Creatinine, µmol/L	71	54–100	-
Glucose, mmol/L	10.25	6.35–17.1	-
LRINEC score	5.5	± 3.06	-

HIV, human immunodeficiency virus; NF, necrotising fasciitis; LRINEC, laboratory risk indicator for necrotising fasciitis.

**FIGURE 1:**
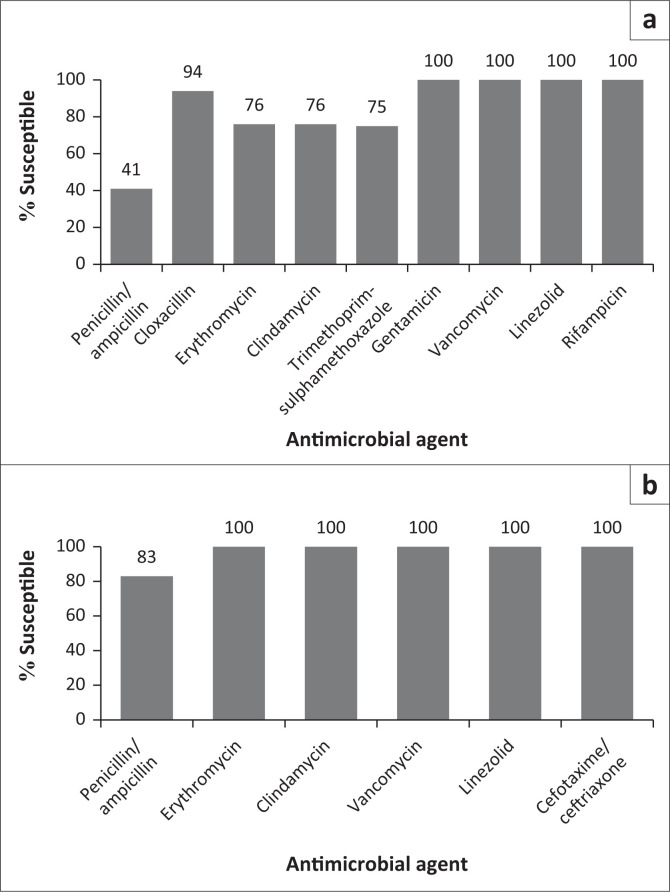
Susceptibilities to selected routinely tested antimicrobials for Gram-positive isolates, 2014–2020, (a) *Staphylococcus aureus* (*n* = 18) and (b) *Streptococci* (*n* = 20).

Microbiology analysis revealed predominately type II (monomicrobial) category (31 [58.49%]) (Table 1). There was a total of 88 first isolates including probable contaminants, which were included in the final analysis. A total of 48 (55%) were Gram-positive bacteria, 39 isolates (44%) were Gram-negative bacteria, and 1 isolate (1%) was *Candida* species. The number of anaerobic microorganisms isolated in the analysis period was 5 (6%). *Staphylococcus aureus* represented the largest proportion of Gram-positive bacteria cultured (18 [38%]), and 17 (94%) was susceptible to cloxacillin (Figure 1). Methicillin resistance was found in 1 case of (6%) *Staphylococcus aureus* (Table 2). There were only 2 (4%) cases of *Streptococcus pyogenes* in the group of Gram-positive bacteria, which were also susceptible to clindamycin.

**TABLE 2:** A summary of the total Gram-positive and Gram-negative isolates, 2014–2020.

Organisms	2014–2020								
	Total first isolates	Total isolates†		Group‡		Group§		Resistent isolates	
		*n*	%	*n*	%	*n*	%	*n*	%
Total isolates	88	-	-	-	-	-	-	-	-
Total Gram-positive bacteria¶	-	48	55	-	-	-	-	-	-
*Staphylococcus aureus*	-	-	-	18	38	-	-	-	-
MRSA	-	-	-	-	-	-	-	1	6
*Streptococci*	-	-	-	20	42	-	-	-	-
Total Gram-negative bacteria††	-	39	44	-	-	-	-	-	-
Total *Enterobacterales*	-	-	-	-	-	30	77	-	-
3GC-resistance	-	-	-	-	-	-	-	4	13
CRE	-	-	-	-	-	-	-	2	7
Total NF-GNB	-	-	-	7	18	-	-	-	-

MRSA: methicillin-resistant *Staphylococcus aureus*; 3GC: Third-generation cephalosporin; NF-GNB: Non-fermenter Gram negative bacilli; CRE: Carbapenem-resistant *Enterobacterales*.

† , Total isolates (Gram-positive, Gram-negative bacteria and *Candida* species) Groups;

‡ , Total Gram-positive bacteria;

§ , Total Gram-negative bacteria;

¶ , Total Gram-positive bacteria includes: *Staphylococcus aureus* (*n* = 18); Coagulase-negative staphylococcus (*n* = 5); *Streptococcus anginosus* (*n* = 5); *Enterococcus* species (*n* = 7); *Peptostreptococcus* species (*n* = 2); *Streptococcus agalactiae* (*n* = 2); *Streptococcus pyogenes* (*n* = 2); *Streptococcus thoraltensis* (*n* = 2); *Streptococcus* species (*n* = 1); *Streptococcus dysgalactiae* (*n* = 1); *Corynebacterium* species (*n* = 2); *Eubacterium limosum* (*n* = 1);

†† , Total Gram-negative bacilli included: *Enterobacterales* [*Escherichia coli* {*n* = 14}; *Klebsiella* species {*n* = 9}; *Enterobacter* species {*n* = 3}; *Citrobacter* species {*n* = 2}; *Proteus* species {*n* = 2}]; NF-GNB [*Pseudomonas* species {*n* = 6}; *Acinetobacter baumannii* {*n* = 1}]; *Bacteroides* species (*n* = 2).

**FIGURE 2:**
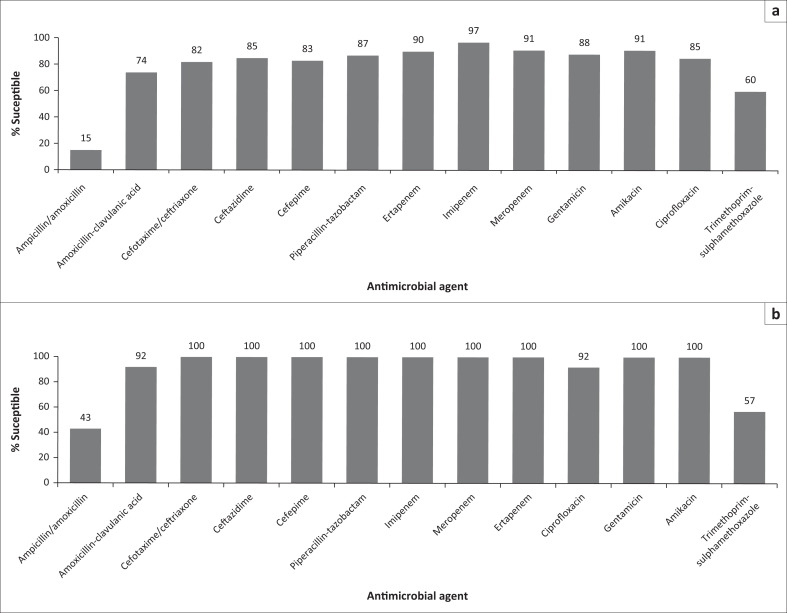
Susceptibilities to routinely tested antimicrobials for Enterobacterales isolates, 2014–2020, (a) *Enterobacterales* (*n* = 30) and (b) *Escherichia coli* (*n* = 14).

*Escherichia coli* was the main species isolated in the Gram-negative bacteria (14 [36%]) with 11 (92%) isolates within the *Enterobacterales* susceptible to amoxicillin and/or clavulanic acid (Figure 2). Multi-drug resistance (MDR) was detected in *Klebsiella pneumoniae* (three isolates) and *Enterobacter* species (one isolate) being resistant up to the third-generation cephalosporins, and carbapenem-resistant *Enterobacterales* (CRE) was detected in two isolates (Table 2). The majority of first isolates (60 [68%]) including *Staphylococcus aureus* and the anaerobes would have been covered by the amoxicillin and/or clavulanic acid and clindamycin used.

The non-fermenter Gram-negative bacteria (NF-GNB) were minimal (7 [8%]) from the total first isolates with 5 (6%) *Pseudomonas aeruginosa* isolates being highly susceptible to most routinely tested antipseudomonal antibiotics (ceftazidime and piperacillin-tazobactam [80% for both] and 100% for meropenem and imipenem). The other isolated NF-GNB (*Acinetobacter baumannii* [*n* = 1] and *Pseudomonas* species [*n* = 1]) demonstrated lower susceptibilities with the aminoglycosides (gentamycin and tobramycin) and the antipseudomonal carbapenems (imipenem and meropenem) being susceptible.

There was only one yeast isolate (*Candida albicans*) identified during the study period. Although there were no susceptibility results reported, there has not been reports of fluconazole resistant *Candida albicans* reported in our institution.

Bivariate analysis between demographics, comorbidity, LRINEC score, and microbiology type of NF showed no statistical significance. However, there was an association between the anatomic site affected by NF and the microbiology type (*p* = 0.03) (Table 3).

**TABLE 3:** Bivariate analysis of characteristics and microbiology type of necrotising fasciitis.

Patient characteristics	Polymicrobial (Type I)			Monomicrobial (Type II)			Fungal (Type IV)			*p* *
	*n*	Median	Mean	*n*	Median	Mean	*n*	Median	Mean	
**Age**	48	38–53	-	44	39–59	-	46	46–46	-	0.97
**Gender**	-	-	-	-	-	-	-	-	-	0.84
Male	13	-	-	21	-	-	1	-	-	-
Female	8	-	-	10	-	-	0	-	-	-
**Comorbidity**	-	-	-	-	-	-	-	-	-	0.24
HIV	4	-	-	11	-	-	1	-	-	-
Diabetes	4	-	-	8	-	-	0	-	-	-
None	13	-	-	12	-	-	0	-	-	-
**Anatomic site**	-	-	-	-	-	-	-	-	-	0.03
Trunk	3	-	-	11	-	-	0	-	-	-
Lower limbs	6	-	-	11	-	-	0	-	-	-
Upper limbs	1	-	-	3	-	-	0	-	-	-
Perineum and scrotum	9	-	-	2	-	-	1	-	-	-
Head and neck	0	-	-	1	-	-	0	-	-	-
Buttock	2	-	-	3	-	-	0	-	-	-
**LRINEC score**	-	-	4.80 ± 2.94	-	-	6.45 ± 3.05	-	-	2.00 ± 0.00	0.91

HIV, Human Immunodeficiency Virus; LRINEC, Laboratory Risk Indicator for Necrotising Fasciitis.

* , Statistically significant (*p* < 0.05).

## Discussion

Necrotising fasciitis is a lethal soft tissue infection that spreads rapidly along the fascial planes and carries a significant mortality rate if not treated promptly.^1^ The demographics of the patients in our study were similar to the global trend previously reported in the literature with mostly male patients, except that our patients were slightly younger than in previous studies, which reported an average age of more than 50 years.^15,16,17^ South Africa is known to suffer from the infamous ‘Quadruple burden of disease’, which includes trauma of which majority of trauma victims are young patients.^18^

Less than 50% of the study population had no comorbidities and only human immunodeficiency virus (HIV) and diabetes mellitus were reported. Other complex comorbidities associated with NF such as liver disease and kidney disease were not reported. Diabetes mellitus is the commonly reported comorbidity associated with NF across the world.^2,15,19^ Al-Qurayshi et al.^15^ reported that half the patients in a cross-sectional study of 4178 in the United States with NF had diabetes. Diabetic patients have increased susceptibility to infection. In our context, HIV was the most commonly reported comorbidity (30.1%), less so in other African countries such as for example, Kenya (6%).^20^ Apart from the immunosuppression caused by HIV infection, associated organ dysfunction and opportunistic infections may further weaken the already fragile immune system. The HIV population is also more prone to infections that would normally be innocuous to a fully functional immune system.^21^

Type II (monomicrobial) was the common type of NF. *Staphylococcus aureus* represented the largest proportion of the Gram-positive bacteria with a very low rate of methicillin resistance. *Escherichia coli* constituted a great proportion of the Gram-negative microorganism. Although there was a concerning resistance of *Klebsiella pneumoniae* and *Enterobacter* species in the Gram-negative group fortunately, *Klebsiella pneumoniae* and *Enterobacter* species were not common isolates in the NF specimens. In an England cohort study of 11 042 by Bodansky et al.,^19^ a predominance of Gram-positive isolates (mostly were *Staphylococcus aureus*) followed by Gram-negative isolates which were mostly *Escherichia coli* and *Klebsiella pneumoniae* being reported. These findings are similar to our study. Contrary to a Scandinavian multicentre cohort of 409 patients and Malaysian cohort of 469 patients which reported large numbers of Group A *Streptococcus* in the group of Gram-positive bacteria. Group A *Streptococcus* is a virulent microorganism and was associated with septic shock and kidney failure; however, it was not associated with higher mortality.^17,22^ There were only two cases of Group A *Streptococcus* in our study which were 100% sensitive to penicillin and clindamycin. The choice of antimicrobial therapy in the Scandinavian and Malaysian studies were penicillin (Ampicillin and/or sulbactam in Malaysia) and clindamycin.^22,23^ The findings of this study support that amoxicillin and/or clavulanic acid is an appropriate antimicrobial choice for empiric therapy to cover both the commonly isolated Gram-positive and Gram-negative bacteria in our institution. Although *Staphylococcus aureus* was not tested for susceptibility to amoxicillin and/or clavulanic acid, Clinical & Laboratory Standard Institute (CLSI) guidelines considers methicillin (oxacillin) susceptible staphylococci to be susceptible to beta lactam combination agents such as amoxicillin and/or clavulanate, ampicillin and/or sulbactam, and piperacillin and/or tazobactam.^24^ Although Group A *Streptococcus* was not a common isolate in our institution, it is imperative to add clindamycin to the empiric therapy in order to neutralise the virulent exotoxins released by Group A *Streptococ cus*. Adjunctive clindamycin has been proven to reduce mortality and morbidity in Group A *Streptococcus* infections.^25^ Empiric clindamycin can be discontinued as soon as culture results have excluded Group A *Streptococcus* to prevent side effects such as *Clostridioides difficile* infection.

Because of the retrospective nature of study, other predisposing factors could not be explored such as smoking, drug injection, and trauma. As a result of incomplete records, we were not able to determine the risk factors associated with NF. The LRINEC score calculation was also underpowered as there was only about 40% of the patients who had all the parameters required for the score calculation. Patients with complex comorbidities were not studied in this study as they were referred to a tertiary centre. It remains unclear whether they would have a different microbiology profile of NF infections. It is recommended that prospective multicentre studies are conducted to understand NF in details in the South African context.

## Conclusion

In our study we found relatively younger patients, predominantly males with a low LRINEC score with NF affecting mainly the lower limbs. The NF was mostly type II (monomicrobial). *Staphylococcus aureus* and *Escherichia coli* were the most common pathogens cultured in the Gram-positive and Gram-negative groups, respectively. There was an overall low rate of antimicrobial resistance to amoxicillin and/or clavulanic acid, and clindamycin, thus the combination can be used as an empiric therapy.
